# Giant Right Intrathoracic Myxoid Fusocellular Lipoma

**DOI:** 10.1155/2015/302189

**Published:** 2015-10-05

**Authors:** Petre V. H. Botianu, Anda Mihaela Cerghizan, Alexandru M. Botianu

**Affiliations:** ^1^Surgical Clinic 4, M5 Department, University of Medicine and Pharmacy of Tirgu Mures, Gheorghe Marinescu 1, 540139 Tirgu Mures, Romania; ^2^Medical Clinic 3, M3 Department, University of Medicine and Pharmacy of Tirgu Mures, Revolutiei 35, 540043 Tirgu Mures, Romania

## Abstract

Intrathoracic lipomas are rare benign tumors; their behavior is not completely clear and their surgical removal may be challenging. We report a case of a giant right intrathoracic myxoid fusocellular lipoma compressing the lung, tracheobronchial tree, and esophagus which was removed through a posterolateral thoracotomy. Complete removal resulted in resolution of the chest pain and improvement of the dyspnea, with no recurrence at 4-year follow-up.

## 1. Introduction

Lipomas are common benign tumors of mesenchymal origin. They are usually located in the subcutaneous fat and are easy to treat; their surgical removal is usually simple, even for large tumors [1]. Intrathoracic location of this disease is much more rare and difficult to diagnose, and surgical removal may be challenging [2]. We report a patient with a large right intrathoracic lipoma that was completely removed through thoracotomy with complete resolution of the symptoms related to the compressive effect of the tumor.

## 2. Case Report

**Figure 1:**
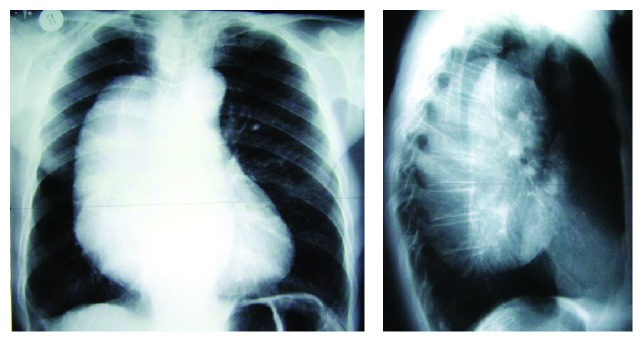
Preoperative chest X-ray showing a large intrathoracic tumor.

**Figure 2:**
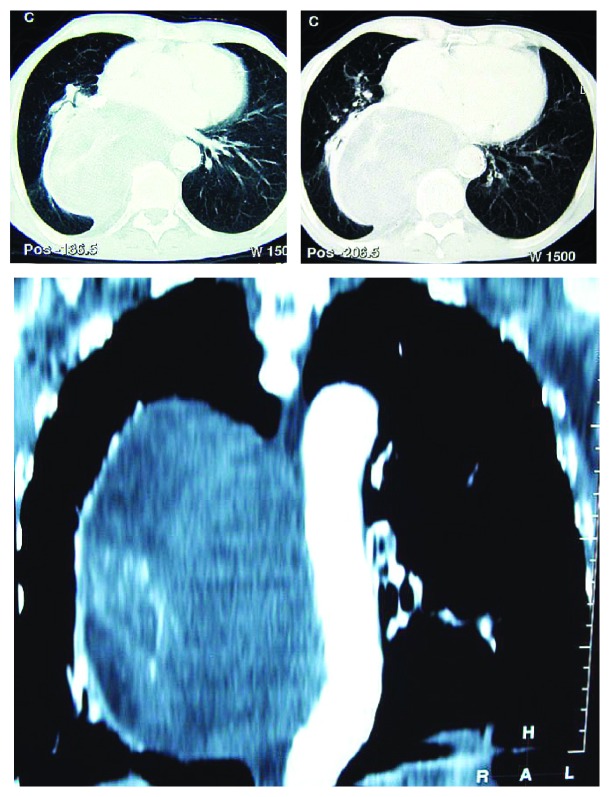
Preoperative CT scan: well-delineated mass with fatty densities and compression on the lung, trachea, right bronchus, and esophagus.

We report a 70-year-old male patient with a history of pleural effusion during childhood, stroke, nasopalpebral basocellular carcinoma excised 5 years before and severe heart disease resulting in NYHA III heart failure. His main actual complaints were pain and worsening dyspnea with no response to medical therapy. Chest X-ray (Figure 1) and CT scan (Figure 2) showed a large tumor located in the right hemithorax, with fatty densities and compressive effect. Bronchoscopy and upper digestive endoscopy showed an extrinsic compression of the trachea and right main bronchus, respectively, and esophagus, but without direct invasion of these structures. Due to the persistent pain and dyspnea the patient was referred to our unit for surgical removal of the tumor.

**Figure 3:**
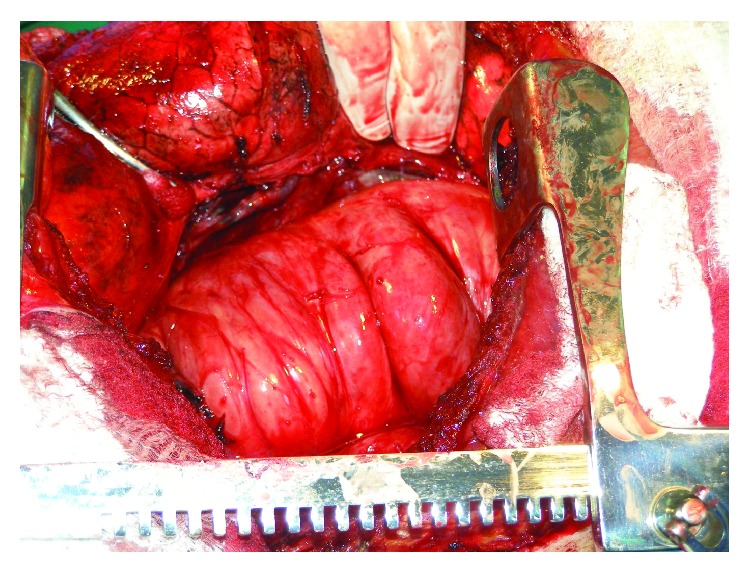
Intraoperative image showing the tumor completely dissected from the lung and covered by the parietal pleura.

Surgery was performed using a large posterolateral thoracotomy. The approach was very difficult due to dense adhesions between the lung and the chest wall (probably secondary to the pleural effusion during childhood). A complete extrapulmonary tumor covered by the parietal pleura was found, with 3 vascular pedicles arising from the posterior intercostal vessels which required separate ligation. There was a cleavage plane that allowed the dissection of the tumor from the trachea, esophagus, and aorta and complete removal of the tumor (Figure 3).

**Figure 4:**
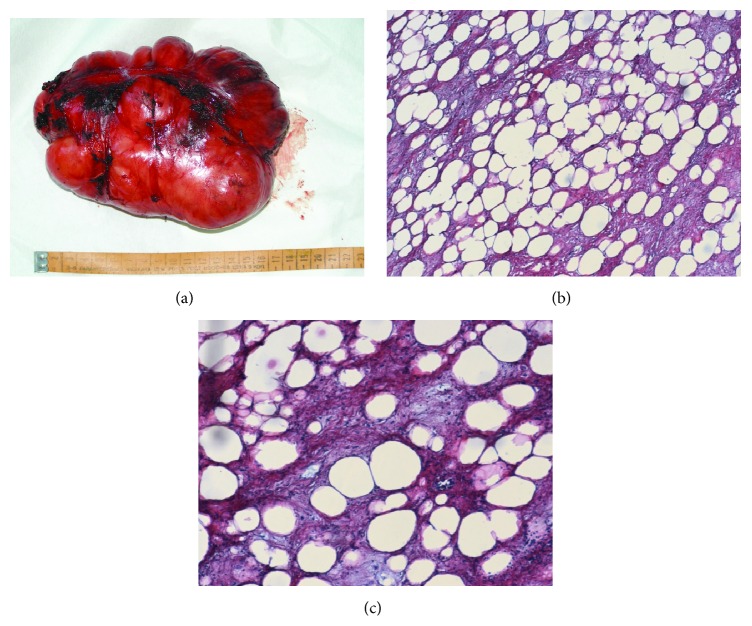
Operative specimen (a) and pathologic examination—hematoxylin-eosin 5x (b) and 10x (c) showing a myxoid spindle cell lipoma.

The operative specimen measured 17 × 10 × 8 cm and weighed 1850 g (Figure 4(a)). Pathologic examination showed a myxoid fusocellular lipoma with no atypia (Figures 4(b) and 4(c)).

The postoperative course was complicated by a bronchopneumonia requiring prolonged antibiotic treatment. There was an obvious improvement of the dyspnea and resolution of the chest pain. At 4-year follow-up, there are no signs of recurrence.

## 3. Discussions

According to their origin, intrathoracic lipomas may be classified as endobronchial, pulmonary, mediastinal (including cardiac), diaphragmatic, and pleural; an hourglass development through the intercostal space is also possible [3, 4]. In our case, due to the large dimensions of the tumor and the multiple adhesions from previous pleural effusion, the origin is not obvious. The fact that after the complete mobilization of the tumor the lung remained free and the presence of blood supply coming from the intercostal vessels strongly suggest a pleural lipoma, arising from the subpleural fatty tissue. Despite the large dimensions, the tumor had only an intrathoracic development.

The modern diagnosis of intrathoracic lipomas is based mainly on CT, which shows a well-delineated tumor with fatty densities [5]. However, there are other fat-containing masses that must be taken into consideration in the differential diagnosis, such as hamartoma, lipoid pneumonia, thymolipoma, lipoblastoma, teratoma, and teratocarcinoma. Most of the aforementioned lesions present with inhomogeneous densities, which allows for an easy differential diagnosis. Malignant lesions often present with an infiltrative aspect, invading the surrounding structures [6, 7]. However, even in the cases with a typical lipoma CT aspect, a malignant component is difficult to exclude (even on biopsy specimens), which is a plea for complete surgical removal of this kind of lesions [2]. For the lesions located near the diaphragm, diaphragmatic hernias and localized eventrations containing omentum, which is a fatty structure, must be also taken into consideration and excluded by careful 3D CT reconstructions or MRI examination [7].

Due to their rarity, the exact behavior of these tumors is not known. In the available literature, we were able to find only case reports and small series. Sakurai et al. emphasize that their clinical-pathological behavior is not always as straightforward as expected, with the possibility of liposarcoma or an infiltrative development [2]. Malignant transformation is a very rare possibility which should be also taken into consideration [8]. The difficult differentiation between benign lipoma and well-differentiated liposarcoma on small biopsy fragments is also a fact that must be taken into consideration as an argument for complete removal [9].

The indication for surgery is a matter of debate. Although it is a benign tumor, most authors advocate surgical removal due to the risks associated with the increasing of the dimensions and possible complications [2, 10, 11]. In our case, the indication for removal was based mainly on the obvious compression of the lung, with persistent dyspnea despite the aggressive medical treatment of the associated heart disease.

In selected cases, smaller tumors may be removed using a minimally invasive approach [12], but this was not the case in our patient.

## 4. Conclusions

Intrathoracic lipomas may represent a challenge despite their benign nature. A careful dissection and an adequate approach allow complete removal even in large tumors, with clinical improvement secondary to the removal of the compression.
